# Supplementary material for: Acute effects of nitrate and breakfast on working memory, cerebral blood flow, arterial stiffness, and psychological factors in adolescents: Study protocol for a randomised crossover trial
Source: PLoS One. 2023 May 19;18(5):e0285581. doi: 10.1371/journal.pone.0285581 (PMC10198498; doi:10.1371/journal.pone.0285581)
Supplement: S1 Checklist — (PDF) [file pone.0285581.s001.pdf]

SPIRIT 2013 Checklist: Recommended items to address in a clinical trial protocol and related documents*

Section/item	Item No	Description	Addressed on page number
**Administrative information**			
Title	1	Descriptive title identifying the study design, population, interventions, and, if applicable, trial acronym	1
Trial registration	2a	Trial identifier and registry name. If not yet registered, name of intended registry	2
	2b	All items from the World Health Organization Trial Registration Data Set	2
Protocol version	3	Date and version identifier	25
Funding	4	Sources and types of financial, material, and other support	26
Roles and responsibilities	5a	Names, affiliations, and roles of protocol contributors	1, 26
	5b	Name and contact information for the trial sponsor	1
	5c	Role of study sponsor and funders, if any, in study design; collection, management, analysis, and interpretation of data; writing of the report; and the decision to submit the report for publication, including whether they will have ultimate authority over any of these activities	26
	5d	Composition, roles, and responsibilities of the coordinating centre, steering committee, endpoint adjudication committee, data management team, and other individuals or groups overseeing the trial, if applicable (see Item 21a for data monitoring committee)	26

**Introduction**			
Background and rationale	6a	Description of research question and justification for undertaking the trial, including summary of relevant studies (published and unpublished) examining benefits and harms for each intervention	4-8
	6b	Explanation for choice of comparators	4-8
Objectives	7	Specific objectives or hypotheses	8-11
Trial design	8	Description of trial design including type of trial (eg, parallel group, crossover, factorial, single group), allocation ratio, and framework (eg, superiority, equivalence, noninferiority, exploratory)	12
**Methods: Participants, interventions, and outcomes**			
Study setting	9	Description of study settings (eg, community clinic, academic hospital) and list of countries where data will be collected. Reference to where list of study sites can be obtained	12
Eligibility criteria	10	Inclusion and exclusion criteria for participants. If applicable, eligibility criteria for study centres and individuals who will perform the interventions (eg, surgeons, psychotherapists)	12
Interventions	11a	Interventions for each group with sufficient detail to allow replication, including how and when they will be administered	14-16
	11b	Criteria for discontinuing or modifying allocated interventions for a given trial participant (eg, drug dose change in response to harms, participant request, or improving/worsening disease)	12
	11c	Strategies to improve adherence to intervention protocols, and any procedures for monitoring adherence (eg, drug tablet return, laboratory tests)	12-13
	11d	Relevant concomitant care and interventions that are permitted or prohibited during the trial	12-16
Outcomes	12	Primary, secondary, and other outcomes, including the specific measurement variable (eg, systolic blood pressure), analysis metric (eg, change from baseline, final value, time to event), method of aggregation (eg, median, proportion), and time point for each outcome. Explanation of the clinical relevance of chosen efficacy and harm outcomes is strongly recommended	17-20
Participant timeline	13	Time schedule of enrolment, interventions (including any run-ins and washouts), assessments, and visits for participants. A schematic diagram is highly recommended (see Figure)	14-16,25; Figure 1

Sample size	14	Estimated number of participants needed to achieve study objectives and how it was determined, including clinical and statistical assumptions supporting any sample size calculations	21
Recruitment	15	Strategies for achieving adequate participant enrolment to reach target sample size	12-13
**Methods: Assignment of interventions (for controlled trials)**			
Allocation:			
Sequence generation	16a	Method of generating the allocation sequence (eg, computer-generated random numbers), and list of any factors for stratification. To reduce predictability of a random sequence, details of any planned restriction (eg, blocking) should be provided in a separate document that is unavailable to those who enrol participants or assign interventions	13
Allocation concealment mechanism	16b	Mechanism of implementing the allocation sequence (eg, central telephone; sequentially numbered, opaque, sealed envelopes), describing any steps to conceal the sequence until interventions are assigned	13
Implementation	16c	Who will generate the allocation sequence, who will enrol participants, and who will assign participants to interventions	13
Blinding (masking)	17a	Who will be blinded after assignment to interventions (eg, trial participants, care providers, outcome assessors, data analysts), and how	13
	17b	If blinded, circumstances under which unblinding is permissible, and procedure for revealing a participant’s allocated intervention during the trial	N/A blinding not possible in this study design.
**Methods: Data collection, management, and analysis**			
Data collection methods	18a	Plans for assessment and collection of outcome, baseline, and other trial data, including any related processes to promote data quality (eg, duplicate measurements, training of assessors) and a description of study instruments (eg, questionnaires, laboratory tests) along with their reliability and validity, if known. Reference to where data collection forms can be found, if not in the protocol	13-20
	18b	Plans to promote participant retention and complete follow-up, including list of any outcome data to be collected for participants who discontinue or deviate from intervention protocols	12

Data management	19	Plans for data entry, coding, security, and storage, including any related processes to promote data quality (eg, double data entry; range checks for data values). Reference to where details of data management procedures can be found, if not in the protocol	20-21
Statistical methods	20a	Statistical methods for analysing primary and secondary outcomes. Reference to where other details of the statistical analysis plan can be found, if not in the protocol	21-23
	20b	Methods for any additional analyses (eg, subgroup and adjusted analyses)	22-23
	20c	Definition of analysis population relating to protocol non-adherence (eg, as randomised analysis), and any statistical methods to handle missing data (eg, multiple imputation)	23
**Methods: Monitoring**			
Data monitoring	21a	Composition of data monitoring committee (DMC); summary of its role and reporting structure; statement of whether it is independent from the sponsor and competing interests; and reference to where further details about its charter can be found, if not in the protocol. Alternatively, an explanation of why a DMC is not needed	N/A No formal committee is needed because a short trial and minimal harms. Study staff (authors) will monitor.
	21b	Description of any interim analyses and stopping guidelines, including who will have access to these interim results and make the final decision to terminate the trial	N/A no interim analyses will be performed
Harms	22	Plans for collecting, assessing, reporting, and managing solicited and spontaneously reported adverse events and other unintended effects of trial interventions or trial conduct	N/A this trial has minimal harm
Auditing	23	Frequency and procedures for auditing trial conduct, if any, and whether the process will be independent from investigators and the sponsor	N/A no auditing is planned
**Ethics and dissemination**			

Research ethics approval	24	Plans for seeking research ethics committee/institutional review board (REC/IRB) approval	26
Protocol amendments	25	Plans for communicating important protocol modifications (eg, changes to eligibility criteria, outcomes, analyses) to relevant parties (eg, investigators, REC/IRBs, trial participants, trial registries, journals, regulators)	Modifications can be found on the trial registry
Consent or assent	26a	Who will obtain informed consent or assent from potential trial participants or authorised surrogates, and how (see Item 32)	26
	26b	Additional consent provisions for collection and use of participant data and biological specimens in ancillary studies, if applicable	N/A no additional consent was needed.
Confidentiality	27	How personal information about potential and enrolled participants will be collected, shared, and maintained in order to protect confidentiality before, during, and after the trial	20-21
Declaration of interests	28	Financial and other competing interests for principal investigators for the overall trial and each study site	26
Access to data	29	Statement of who will have access to the final trial dataset, and disclosure of contractual agreements that limit such access for investigators	20-21
Ancillary and post-trial care	30	Provisions, if any, for ancillary and post-trial care, and for compensation to those who suffer harm from trial participation	26
Dissemination policy	31a	Plans for investigators and sponsor to communicate trial results to participants, healthcare professionals, the public, and other relevant groups (eg, via publication, reporting in results databases, or other data sharing arrangements), including any publication restrictions	26
	31b	Authorship eligibility guidelines and any intended use of professional writers	27
	31c	Plans, if any, for granting public access to the full protocol, participant-level dataset, and statistical code	27
**Appendices**			
Informed consent materials	32	Model consent form and other related documentation given to participants and authorised surrogates	Available on request

Biological specimens	33	Plans for collection, laboratory evaluation, and storage of biological specimens for genetic or molecular analysis in the current trial and for future use in ancillary studies, if applicable	20

*It is strongly recommended that this checklist be read in conjunction with the SPIRIT 2013 Explanation & Elaboration for important clarification on the items. Amendments to the protocol should be tracked and dated. The SPIRIT checklist is copyrighted by the SPIRIT Group under the Creative Commons "Attribution-NonCommercial-NoDerivs 3.0 Unported" license.

Preliminary project plan: Acute effects of nitrate supplementation with breakfast on cognition and vascular health and the effects of breakfast on cognition and mood in adolescents in a randomised crossover design

Background:

Inorganic nitrate (NO_3^-) can be obtained from the diet, nitrate rich sources include: rocket (rucola), spinach, lettuce and beetroot (1) with green leafy vegetables contributing to the majority of nitrate consumption (2). Intake of nitrate-rich foods has previously been found to increase cerebral blood flow (CBF) and stimulate cerebral perfusion in the pre-frontal cortex; which is the part of the brain associated with executive function and working memory (3). In a relatively recent intervention study dietary nitrate from beetroot juice was found to improve cognitive performance on a serial-3 subtraction cognition task that assesses working memory in healthy adults and was found to modulate CBF during cognitive performance tasks (4). Dietary nitrate is converted to nitrite in the oral cavity, which is further reduced in the stomach to nitric oxide (NO) (5). Nitric oxide has been shown to function as a vasodilator and reduced availability and functionality of NO in the body has been associated with endothelial dysfunction and cardiovascular disease (CVD) (6-8). The effects of improved cognitive performance from nitrate could be in part due to increased CBF and improved coupling of blood flow with metabolism and/or higher NO mediated activity (9). Despite this, mixed results exist from randomised trials in adults on whether dietary nitrate consumption has an effect on cognitive function and CBF (3).

The consumption of dietary nitrate is also associated to multiple vascular health benefits and the vascular effects of nitrate is too linked with increased nitrite and NO levels (10) through the enterosalivary nitrate-nitrite-NO pathway (11). A recent systematic review including only experimental studies in adults found that intake of inorganic nitrate reduced blood pressure and arterial stiffness (pulse wave velocity (PWV) and augmentation index (AI)) (5); with arterial stiffness being an independent predictor of cardiovascular risk (12). However, no studies, to our knowledge have investigated the effects of nitrate on vascular health in adolescents.

A more widely studied topic in children and adolescents is the effects of breakfast consumption on cognitive function (CF), with a recent systematic review finding 24 studies comparing the effects of breakfast and breakfast omission in these populations (13). Breakfast consumption has been suggested to be linked with numerous benefits to overall health and well-being in adolescents (13). Adolescents that habitually consume breakfast are more likely to have a higher diet quality (14) and have a decreased risk of being overweight and obese (15). Despite this, the prevalence of breakfast skipping in children and adolescents across the United States and Europe has been shown to be between 10-30% (16) and between 27-32% in Swedish adolescents (17). Moreover, Adolphus et al, 2016[(13)] found breakfast consumption compared to fasting to have positive, same morning effects on CF's, including: attention, executive functioning and memory in intervention studies involving children and adolescents. Results from an intervention study in adolescents showed that the consumption of a low glycaemic index breakfast led to improved response times and accuracy compared to breakfast omission in a range of CF tasks including attention and memory cognitive domains, especially in more demanding cognitive tasks (18).

The consumption of breakfast compared to breakfast omission has also been found to have positive effects on adolescents' mood. Cooper et al, 2011[19] found that breakfast consumption led to higher self-reports of energy and fullness as well as lower self-reports on tiredness in adolescents. Additionally, in adolescents who habitually skip breakfast, higher self-reports of alertness and contentment in those that consumed breakfast compared to breakfast omission were reported (20). Thus it is suggested that consuming breakfast can influence and play a significant role in mood and alertness in adolescents.

The acute effects of nitrate with breakfast on cognition, vascular health and mood is believed to not have been conducted before in adolescents nor has the acute effects of breakfast on cognition, CBF and mood been carried out in Swedish adolescents. Thus there is a research gap in the field to study these topics in adolescents and a need to investigate the effects of nitrate on CF and CBF. Consuming nitrate-rich vegetables with breakfast is considered to be an achievable, ecologically valid and relatively low-cost method to increase consumption of dietary nitrate in adolescents. Despite this, adherence to nitrate-rich vegetables could be a challenge as previous research has found the consumption fruit and vegetables amongst Swedish adolescents to be below 50% of the recommended intake (21).

The work of this study will be useful to assess the effects that nitrate with breakfast has on cognition, CBF and mood as well as to see if the same acute effects of dietary nitrate intake on vascular health as seen in adults also occurs in adolescents. Results could indicate the importance of consuming breakfast and nitrate-rich vegetables for improving adolescent's cognitive ability and positive emotions of mood, thus having important implications for academic performance and achievement. The consumption of nitrate-rich vegetables could also lead to benefits of vascular health in this young population and thus have the potential to reduce future cardiovascular health problems.

Research aim and questions:

The main aim of this study is to assess the effects of breakfast supplemented with nitrate on measures of cognition in 13–15-year-old adolescents.

The secondary aims of this study is to investigate the effects of breakfast compared to breakfast omission on measures of mood and the effects of breakfast supplemented with nitrate on arterial stiffness and CBF.

Research questions:

Primary question: What effect does breakfast supplemented with nitrate compared to a standardised breakfast and breakfast omission have on:

a. Cognitive performance
b. Mood

Secondary questions:

1. Does breakfast supplemented with nitrate have acute effects on arterial stiffness and CBF compared to breakfast without nitrate and breakfast omission?
2. Are there significant differences in cognition and mood between breakfast consumption and breakfast omission?

Hypotheses:

1. **1.** Cognitive performance will be significantly higher in the breakfast supplemented with nitrate compared to the standardised breakfast group.
2. **2.** Scores for positive mood and alertness will be significantly higher in the breakfast group compared to the breakfast omission group.
3. **3.** The breakfast supplemented with nitrate group will have significantly lower measures of arterial stiffness and changes in CBF will be significantly different compared to the standardised breakfast group and breakfast omission group.

Project description

Study population:

Adolescents between the ages of 13-15 will be included in the study. Participants will need to have proof of having a double vaccination against covid-19. Participants will also need to understand Swedish. Those that have been diagnosed with diabetes, epilepsy, vascular health conditions/circulatory abnormalities, visual/auditory impairments or those receiving treatment from depression, sleep disorders, psychosis or other psychiatric conditions will be excluded.

Familiarisation:

The researchers will visit the schools that are involved prior to randomization and will introduce all the procedures that will be carried out on the test days to the participants. This will include practising the cognitive tests (as to reduce practice effects) and be familiarised with the mood questionnaire/scales that they will complete. Demographic data and other data for important measures will also be collected, these will include: age, sex, height, weight, head circumference (for fNIRS cap size), food allergies or any dietary intolerances and a health questionnaire. Additionally a short questionnaire will be provided to the participants', asking them how often they consume various nitrate-rich foods (pictures of foods will be provided) and how often they eat breakfast. This is to assess the proportion of regular consumers of nitrate-rich foods and the proportion of those that habitually consume breakfast.

Ethics:

Consent from both the participants and their guardians will be collected and required before any experimental and familiarisation tests are carried out. A gift card shall be provided upon participation and all travel arrangements to and from GIH will be arranged with taxi services.

Pre-experimental requisites:

The participants will be asked to fast after they have eaten their dinner on the pre-test day. They will also be asked to eat the same dinner before each test day and will record this meal in a food diary along with any drinks they have consumed. The pre-test day dinner can be of the participants choosing; however a list of nitrate rich foods will be given to the participants and on pre-test days participants will be asked to not consume these foods. Participants will also be asked not to consume any caffeine on experimental test days and to not undergo any moderate to vigorous exercise on pre-test days on the mornings of test days. The participants will have their physical activity and sedentary behaviour monitored (objectively using accelerometers) on the pre-test day and be asked to complete a physical activity, diet and sleep diary on pre-test days to standardize these measures for test-days.

Intervention arms:

All trials will be performed at GIH and upon arrival to the laboratory, details of the pre-experimental requisites will be collected. This study will be a cross-over randomised trial with three study arms:

1. No breakfast
2. Low nitrate and low glycaemic breakfast (control)
3. High nitrate and low glycaemic breakfast; nitrate will be provided through green leafy vegetables: rocket or spinach

On the first test-day visit participants will be able to choose how much food they want to consume from the foods provided. On subsequent visits the same and exact amount of food will be given to the participants, of course no breakfast will be provided when participants are in the no breakfast intervention group. Every group, however, will drink water ad libitum through the course of the experimental days. We do not want the participants to go hungry when they are in the experimental breakfast arms and we would like breakfast consumption to be as close to the participants habitual diet as possible, hence why participants can choose the amount of breakfast that they deem will satisfy them. The breakfast provided will be of low glycaemic index to reduce the effects of glucose as we are interested in the acute effects of nitrate. The amount of nitrate provided will be the same for each participant and they will be asked to eat all of the nitrate rich food source provided (either rocket or spinach). In the scenario that the participant does not consume all the nitrate food source, then the amount they consumed will be documented and the mean nitrate intake will be calculated. This standardised amount of nitrate is still to be decided but will not exceed the acceptable daily intake of 3.7 mg nitrate kg^{-1} body weight day^{-1} (22), and samples will be taken from the nitrate-rich vegetable to assess nitrate content before consumption by participants.

There will be a 80 minute sitting still period in-between measurements, where the participants will be able to read books or listen to audio books that can be related to their schoolwork, but not be allowed to use any technological devices nor partake in any physical activity. A social break will be provided, whereby the participant can talk to one of the researchers. There will be a minimum of 7 days washout period in-between each visit of each participant.

Outcome measures:

Primary outcome: Cognition

Secondary outcomes: Mood, arterial stiffness, CBF and nitrate concentrations

Cognitive performance

This will be measured through working memory ability from the n-back task consisting of 1, 2 and 3-back tests (23). A computerised numerical version of the n-back will be used, participants shall indicate if the digit presented on the screen was the same digit that was presented 1 digit ago (1-back), 2 digits ago (2-back) or three digits ago (3-back). Participants will indicate this via a key press within a 2-second period from the onset of the digit, whereby each digit is presented for 1.5 seconds with an interstimuli interval of 500 milliseconds (ms) (pseudo randomisation for cognitive tasks will be used). The cognitive tests will be pseudo randomised. E-Prime 2.0 (Psychology Software Tools) was used to create the n-back tests and this software will be used for all cognitive tests. Average reaction time (m/s) and accuracy (number of correct responses) will be measured for cognitive performance for each task

across 2 blocks of 20-digit sequences (1-back) or 4 blocks of 20-digit sequences (2- and 3-back).

Mood

Participants' mood and subjective feelings will be recorded using the Positive and Negative Affect Scale (PANAS) (24). PANAS has been preliminarily validated in adolescents and shown to be a reliable measure of positive and negative moods (25) and has also been used in Swedish adolescents before (26). Whilst alertness will be measured using a visual analogue scale (VAS) (27) and sleepiness using the Karolinska Sleepiness Questionnaire (28). We are particularly interested in how alert/sleepy the participants feel after breakfast consumption and breakfast omission which may have important implications for school performance and achievement.

Arterial stiffness

Arterial stiffness will be measured as augmentation index (AI) and pulse wave velocity (PWV) using SphygmoCor XCEL PWA/PWV system (29). The technology is a non-invasive, reproducible, and accurate method that uses waveform shape parameters from the radial artery to acquire central aortic blood pressure waveform (29). The SphygmoCor XCEL PWA/PWV system has been tested for validity in this age group (30).

The aortic pressure waveform shall be derived from the radial waveform through a validated transfer function (31). 5 minutes after of supine rest, high fidelity pressure waveforms will be recorded (32), these will be expressed as AI (percent). PWV will be assessed between carotid and femoral arteries and expressed as velocity (m/s).

Cerebral blood flow (CBF)

CBF will be measured by changes in hemoglobin (oxy-Hb) using a non-invasive multi-channel continuous wave fNIRS instrument (portable NIRSport, 8-8 system, with short-separation channels, NIRx Medizintechnik GmbH, Berlin, Germany). The optodes for this instrument will be placed over the pre-frontal cortex, data will be sampled at 7.81 Hz at wavelengths 750 nm and 820 nm. The fNIRS cap has 16 channels created by 8 LED light sources and 8 detectors that are placed according to the standard 10-20 system, with a source-detector separation distance of 3 cm for long-separation channels. Additional short-separation optodes with a distance of 0.8 cm (NIRx Medizintechnik GmbH, Berlin, Germany) to account for superficial blood flow (33) will also be used. CBF was chosen to be measured in the pre-frontal cortex because working memory tests such as the n-back task and serial-subtraction tasks activate this region (33-35). System calibration will be performed before each assessment using NIRStar 15.2 software and using the predefined montage. The fNIRS signals will be visually quality checked during data collection for motion artefacts. CBF will be measured whilst participants complete the cognitive tests We will focus on changes in oxy-Hb during the cognitive tasks between the three different trial groups. Deoxygenated (deoxy-Hb) and total hemoglobin (tHb) concentrations will also be recorded.

Nitrate concentrations

Nitrate concentrations will be collected via four saliva samples; one pre-experiment, two mid-experiment and one late experiment. These samples will be centrifuged and stored at -80°C, until the time of analysis (<6 months from collection).

Sample size

To the best of our knowledge no studies have investigated the acute effects of nitrate consumption and vascular health as well as cognitive measures in adolescents. Arterial stiffness and CBF mechanistic outcomes have less variance than cognition and mood, thus the sample size was calculated from effect sizes cognition and mood outcomes. Effect sizes were calculated on G*Power software (Franz Faul, Universität Kiel, Germany, v 3.1.9.2) using differences between two dependent means and standard deviations. The sample size was calculated: using $\alpha = 0.05$, $\beta = 0.8$, correlation = 0.5 and assuming a two-tailed test. The effect size based on the effects of nitrate on working memory cognition = 0.63, giving a sample size = 22. The effect size based on effects of breakfast in mood ranged from 0.67 to 0.48, giving a sample size range between 20 and 36. Our sample size will thus be the largest found here plus 7 to account for 20% drop out rate, thus n = 43.

Data analysis

Two-way repeated measures ANOVA for treatment and time interactions will be used to assess significant differences in arterial stiffness and cognitive/mood outcomes, and with the use of Bonferonni corrections for multiple comparisons. Random mixed effects models with random intercepts will be used to assess differences in CBF between groups. SPSS version 27 and NIRSToolboxAnalyzer will be used for statistical analyses.

References

1. Hord NG, Tang YP, Bryan NS. Food sources of nitrates and nitrites: the physiologic context for potential health benefits. American Journal of Clinical Nutrition. 2009;90(1):1-10.
2. Ma LS, Hu L, Feng XY, Wang SL. Nitrate and Nitrite in Health and Disease. Aging and Disease. 2018;9(5):938-45.
3. Clifford T, Babateen A, Shannon OM, Capper T, Ashor A, Stephan B, et al. Effects of inorganic nitrate and nitrite consumption on cognitive function and cerebral blood flow: A systematic review and meta-analysis of randomized clinical trials. Critical Reviews in Food Science and Nutrition. 2019;59(15):2400-10.
4. Wightman EL, Haskell-Ramsay CF, Thompson KG, Blackwell JR, Winyard PG, Forster J, et al. Dietary nitrate modulates cerebral blood flow parameters and cognitive performance in humans: A double-blind, placebo-controlled, crossover investigation. Physiology & Behavior. 2015;149:149-58.
5. Jackson JK, Patterson AJ, MacDonald-Wicks LK, Oldmeadow C, McEvoy MA. The role of inorganic nitrate and nitrite in cardiovascular disease risk factors: a systematic review and meta-analysis of human evidence. Nutrition Reviews. 2018;76(5):348-71.
6. Li HG, Forstermann U. Nitric oxide in the pathogenesis of vascular disease. Journal of Pathology. 2000;190(3):244-54.
7. Kleinbongard P, Dejam A, Lauer T, Jax T, Kerber S, Gharini P, et al. Plasma nitrite concentrations reflect the degree of endothelial dysfunction in humans. Free Radical Biology and Medicine. 2006;40(2):295-302.
8. Landmesser U, Drexler H. Endothelial function and hypertension. Current Opinion in Cardiology. 2007;22(4):316-20.
9. Toda N, Ayajiki K, Okamura T. Cerebral blood flow regulation by nitric oxide: recent advances. Pharmacol Rev. 2009;61(1):62-97.
10. Bondonno CP, Yang XB, Croft KD, Considine MJ, Ward NC, Rich L, et al. Flavonoid-rich apples and nitrate-rich spinach augment nitric oxide status and improve

endothelial function in healthy men and women: a randomized controlled trial. Free Radical Biology and Medicine. 2012;52(1):95-102.
11. Webb AJ, Patel N, Loukogeorgakis S, Okorie M, Aboud Z, Misra S, et al. Acute blood pressure lowering, vasoprotective, and antiplatelet properties of dietary nitrate via bioconversion to nitrite. Hypertension. 2008;51(3):784-90.
12. Van Bortel LM, Laurent S, Boutouyrie P, Chowienczyk P, Cruickshank JK, De Backer T, et al. Expert consensus document on the measurement of aortic stiffness in daily practice using carotid-femoral pulse wave velocity. Journal of Hypertension. 2012;30(3):445-8.
13. Adolphus K, Lawton CL, Champ CL, Dye L. The Effects of Breakfast and Breakfast Composition on Cognition in Children and Adolescents: A Systematic Review. Advances in Nutrition. 2016;7(3):590S-612S.
14. Deshmukh-Taskar PR, Nicklas TA, O'Neil CE, Keast DR, Radcliffe JD, Cho S. The Relationship of Breakfast Skipping and Type of Breakfast Consumption with Nutrient Intake and Weight Status in Children and Adolescents: The National Health and Nutrition Examination Survey 1999-2006. Journal of the American Dietetic Association. 2010;110(6):869-78.
15. de la Hunty A, Gibson S, Ashwell M. Does Regular Breakfast Cereal Consumption Help Children and Adolescents Stay Slimmer? A Systematic Review and Meta-Analysis. Obesity Facts. 2013;6(1):70-85.
16. Rampersaud GC, Pereira MA, Girard BL, Adams J, Metzl JD. Review - Breakfast habits, nutritional status, body weight, and academic performance in children and adolescents. Journal of the American Dietetic Association. 2005;105(5):743-60.
17. Vereecken C, Dupuy M, Rasmussen M, Kelly C, Nansel TR, Al Sabbah H, et al. Breakfast consumption and its socio-demographic and lifestyle correlates in schoolchildren in 41 countries participating in the HBSC study. International Journal of Public Health. 2009;54:180-90.
18. Cooper SB, Bandelow S, Nute ML, Morris JG, Nevill ME. Breakfast glycaemic index and cognitive function in adolescent school children. British Journal of Nutrition. 2012;107(12):1823-32.
19. Cooper SB, Bandelow S, Nevill ME. Breakfast consumption and cognitive function in adolescent schoolchildren. Physiology & Behavior. 2011;103(5):431-9.
20. Defeyter MA, Russo R. The effect of breakfast cereal consumption on adolescents' cognitive performance and mood. Frontiers in Human Neuroscience. 2013;7.
21. Lemming EW ML, Sipinen JP and AK Lindroos. Riksmaten Ungdom 2016-2017, Så äter ungdomar i Sverige: Livsmedelsverket; 2018 [Available from: https://www.livsmedelsverket.se/globalassets/publikationsdatabas/rapporter/2018/2018-nr-14-riksmatenungdom-huvudrapport_del-1-livsmedelskonsumtion.pdf.
22. Speijers GJAavdB, P.A. Nitrate (and potential endogenous formation of N-nitroso compounds)2003 2021-11-23; Series 50.
23. Kirchner WK. Age differences in short-term retention of rapidly changing information. J Exp Psychol. 1958;55(4):352-8.
24. Crawford JR, Henry JD. The positive and negative affect schedule (PANAS): Construct validity, measurement properties and normative data in a large non-clinical sample. British Journal of Clinical Psychology. 2004;43:245-65.
25. Huebner ES, Dew T. PRELIMINARY VALIDATION OF THE POSITIVE AND NEGATIVE AFFECT SCHEDULE WITH ADOLESCENTS. Journal of Psychoeducational Assessment. 1995;13(3):286-93.

26. Garcia D, Moradi S. The Affective Temperaments and Well-Being: Swedish and Iranian Adolescents' Life Satisfaction and Psychological Well-Being. Journal of Happiness Studies. 2013;14(2):689-707.
27. Monk TH. A VISUAL ANALOG SCALE TECHNIQUE TO MEASURE GLOBAL VIGOR AND AFFECT. Psychiatry Research. 1989;27(1):89-99.
28. Putilov AA, Donskaya OG. Construction and validation of the EEG analogues of the Karolinska sleepiness scale based on the Karolinska drowsiness test. Clinical Neurophysiology. 2013;124(7):1346-52.
29. Hwang MH, Yoo JK, Kim HK, Hwang CL, Mackay K, Hemstreet O, et al. Validity and reliability of aortic pulse wave velocity and augmentation index determined by the new cuff-based SphygmoCor Xcel. Journal of Human Hypertension. 2014;28(8):475-81.
30. Stabouli S, Printza N, Zervas C, Dotis J, Chrysaidou K, Maliahova O, et al. Comparison of the SphygmoCor XCEL device with applanation tonometry for pulse wave velocity and central blood pressure assessment in youth. Journal of Hypertension. 2019;37(1):30-6.
31. Pauca AL, O'Rourke MF, Kon ND. Prospective evaluation of a method for estimating ascending aortic pressure from the radial artery pressure waveform. Hypertension. 2001;38(4):932-7.
32. Nelson MR, Stepanek J, Cevette M, Covalciuc M, Hurst RT, Tajik AJ. Noninvasive Measurement of Central Vascular Pressures With Arterial Tonometry: Clinical Revival of the Pulse Pressure Waveform? Mayo Clinic Proceedings. 2010;85(5):460-72.
33. Yucel MA, Selb J, Aasted CM, Petkov MP, Becerra L, Borsook D, et al. Short separation regression improves statistical significance and better localizes the hemodynamic response obtained by near-infrared spectroscopy for tasks with differing autonomic responses. Neurophotonics. 2015;2(3).
34. Owen AM, McMillan KM, Laird AR, Bullmore ET. N-back working memory paradigm: A meta-analysis of normative functional neuroimaging. Human Brain Mapping. 2005;25(1):46-59.
35. Kazui H, Kitagaki H, Mori E. Cortical activation during retrieval of arithmetical facts and actual calculation: A functional magnetic resonance imaging study. Psychiatry and Clinical Neurosciences. 2000;54(4):479-85.
